# Psychometric properties of the FACT-G quality of life scale for family caregivers of cancer patients

**DOI:** 10.1007/s11136-020-02477-7

**Published:** 2020-04-03

**Authors:** Lixin Song, Xianming Tan, Jason Bredle, Antonia V. Bennett, Laurel Northouse

**Affiliations:** 1 School of Nursing, University of North Carolina-Chapel Hill, Carrington Hall, Chapel Hill, NC 27599-7460 USA; 2 UNC Lineberger Comprehensive Cancer Center, Chapel Hill, NC USA; 3 UNC Gillings School of Global Public Health, Chapel Hill, NC USA; 4 FACIT.org, 151 Bay Cove Drive, Ponte Vedra, FL USA; 5 School of Nursing, University of Michigan-Ann Arbor, Ann Arbor, MI USA

**Keywords:** Cancer, Caregiver, Quality of life, Reliability, Validity, Psychometric properties

## Abstract

**Purpose:**

This study aimed to examine psychometric properties of a caregiver version of the well-established Functional Assessment of Cancer Therapy-General Scale (FACT-G) after conducting focus groups and obtaining expert input.

**Methods:**

We made minor wording modifications to the Patient FACT-G to enable caregivers to report how the illness affected their overall quality of life (QOL) and well-being on four subscales (physical, social, emotional, functional). We tested the acceptability, precision, factor structure, reliability and validity of the Caregiver FACT-G among partners of prostate cancer patients (*N* = 263) and caregivers (spouses, siblings, adult children) of patients with advanced cancer (breast, lung, colorectal, prostate) (*N* = 484) using data from two Randomized Clinical Trials (RCTs).

**Results:**

With a factor structure similar to the Patient FACT-G, Caregiver FACT-G was acceptable and precise in measuring caregiver QOL, with high inter-factor correlations and internal consistency reliability (Cronbach’s alphas 0.81–0.91). The Caregiver FACT-G had strong convergent validity demonstrated by significant positive correlations with caregiver self-efficacy (0.25–0.63), dyadic communication (0.18–0.51), and social support (0.18–0.54) in both samples. It also had strong discriminant validity evidenced by significant inverse correlations with negative appraisal of caregiving (− 0.37 to − 0.69), uncertainty (− 0.28 to − 0.53), hopelessness (− 0.25 to − 0.60), and avoidant coping (− 0.26 to − 0.58) in both samples. Caregivers’ baseline FACT-G scores were significantly associated with their physical (0.23) and mental well-being (0.54; 4-month follow-up) and their depression (− 0.69; 3-month follow-up), indicating strong predictive validity.

**Conclusion:**

This is the first study evaluating the psychometric properties of the Caregiver FACT-G. More psychometric testing is warranted, especially among caregivers of diverse sociocultural backgrounds.

## Introduction

Caring for a person with cancer is a challenging experience for family caregivers [1, 2]. As the demands of caregiving increase, caregivers report higher distress and lower quality of life (QOL) [3–5]. Cancer patients’ and their caregivers’ responses to illness are interdependent [6, 7]; each person affects the other [8]. Hence, it is essential to assess the QOL of cancer patients and their caregivers so that dyadic, psycho-educational interventions can be developed to address the needs of both members of the dyad (i.e., pair).

Although studies have assessed cancer patients’ QOL [5], few have assessed caregivers’ QOL [9]. Furthermore, caregiver QOL has been measured in multiple ways, making it difficult to compare caregivers’ QOL across studies. For example, caregiver QOL has been measured with *caregiver-focused instruments* (e.g., Caregiver Quality of Life Index-Cancer [10–12]; and Quality of Life Scale-Family Version [13]); with *general health instruments* (e.g., Medical Outcomes Study Health Survey (36-item or 12-item version) [3, 14, 15]) and with instruments that measure a *specific component of QOL* such depression (e.g., Beck Depression Inventory or Center for Epidemiologic Study-Depression instrument [10, 16]). A need remains for a cancer-specific, multi-dimensional instrument to assess the QOL of both patients and their caregivers individually and jointly as a dyad. Parallel versions of the same instrument would make it possible to examine the concordance of patients’ and caregivers’ perceptions of their cancer survivorship experiences and QOL. Dyadic assessments of patient and caregiver QOL, using parallel versions of the same instrument, can help clinicians determine how the QOL of one member of the dyad affects the QOL of the other member (e.g., partner effects). In addition, parallel versions will facilitate development of dyadic interventions to improve QOL for both patients and caregivers.

### Background on the development of the caregiver FACT-G

The Functional Assessment of Cancer Therapy-General (FACT-G; v. 4) [17] has been used widely to measure patient QOL [18]. Thus, in 1995 and with permission from FACIT.org, we began development of a parallel version for caregivers (Caregiver FACT-G) because no caregiver QOL measure was available at that time. The development of the Caregiver FACT-G was based on Lazarus and Folkman’s Transactional Theory of Stress [19] that postulates that individuals who face a potentially threatening event (e.g., cancer treatment) assess the degree of threat and their resources to cope with the event, and then reappraise their situation as either more or less threatening. The theory also contends that this process can affect health outcomes such as QOL [20]. To help develop the Caregiver FACT-G, we also conduced a series of preliminary studies on the psychosocial adjustment of breast and colon cancer patients and their caregivers (i.e., dyads) [21–24].

Our findings indicated that both patients and caregivers reported higher emotional distress than the general population, and they had problems carrying out family, social, and work roles [22]. Furthermore, findings from our longitudinal comparative study found that breast cancer patients and their caregivers had significantly more psychosocial adjustment problems (e.g., decreased marital and family functioning, more uncertainty) over time than did dyads coping with benign breast disease [23]. Next, we conducted two qualitative studies to learn how cancer patients and caregivers perceived their QOL (physical, psychological, and social/family health) [25, 26]. Results from our focus group data indicated that patients and caregivers reported ongoing problems (e.g., managing symptoms and treatment side effects, emotional distress) as well as family, social, and role changes that negatively affected their QOL.

Based on these preliminary findings, we obtained expert feedback from three PhD researchers (with expertise in cancer, caregiving, and the Transactional Theory of Stress) to modify the wording of the patient FACT-G so that caregivers could report on their own QOL as they cared for patients with cancer. We pilot tested the Caregiver FACT-G with three caregivers of women with recurrent breast cancer. They were able to understand the measures and complete it in 3–4 minutes without difficulty (Unpublished work for a grant submitted to American Cancer Society, PI: Northouse). We also piloted the Caregiver FACT-G with five spouse caregivers of men with prostate cancer; they reported no difficulty understanding or completing the questionnaire (Unpublished work for grant R01CA090739; PI: Northouse). We examined the internal consistency of the Caregiver FACT-G in a sample of caregivers (*N* = 189) of women with recurrent breast cancer and found it was very good (0.87). We also tested concurrent validity of the scale, using the Medical Outcomes Study (MOS) SF-36, in this same sample of caregivers. We found significant correlations between Caregiver FACT-G total scores and the MOS SF-36 mental (*r* = 0.62, *p* < 0.05) and physical (*r* = 0.25, *p* < 0.05) dimensions [27].

In the present study, we conducted a comprehensive assessment of the psychometric properties of the Caregiver FACT-G using data from two large randomized controlled trials (RCT) [28, 29]. This study aimed to examine the acceptability and precision, factor structure, reliability and construct and predictive validity of the Caregiver FACT-G. In this way, results would be available to researchers and clinicians interested in using the Caregiver FACT-G. Based on the Transactional Theory of Stress [19, 30], we expected that higher caregiver self-efficacy, social support, patient-caregiver dyadic communication, and use of active coping would be related to better caregiver QOL (i.e., convergent validity). In addition, we anticipated that more negative appraisal of caregiving, uncertainty, hopelessness, and use of avoidant coping would be related to lower caregiver QOL [7, 30] (discriminant validity). Finally, we hypothesized that caregivers with higher FACT-G scores at baseline would report better physical and mental well-being and lower depression at follow-up 3–4 months later (predictive validity).

## Method

We conducted secondary analyses of data from two RCTs [28, 29] that tested the efficacy of a psycho-educational program (FOCUS: Family Involvement, Optimistic Attitude, Coping Effectiveness, Uncertainty Reduction, and Symptom Management) on patient and caregiver QOL and other psychosocial outcomes. FOCUS is a psycho-educational, home-based, dyadic intervention that provided information and support to cancer patients and their family caregiver. FOCUS was delivered using a series of home visits and phone calls to patient-caregiver dyads by an advanced practice nurse over a three-month period of time [31]. The first RCT, which we refer to as the “Prostate RCT”, was conducted among men with prostate cancer and their partners (*N* = 263 dyads) who were randomized as a pair to either usual care or the 5-session FOCUS Program [28]. The second RCT, the “Advanced Cancer RCT”, was conducted among patients with advanced lung, colorectal, prostate, or breast cancer and their caregivers (*N* = 484 dyads) who were randomized as a pair to: usual care, a 3-session FOCUS Program, or a 6-session FOCUS Program [29].

To eliminate the effects of FOCUS on QOL, we used the caregivers’ baseline data (T1) prior to randomization in both RCTs to assess the Caregiver FACT-G’s acceptability, precision, factor structure, reliability, and construct validity (convergent and discriminant). We used control group data from the first follow-up (T2) to assess Caregiver FACT-G’s predictive validity.

### Participants

Patient-caregiver dyads in the Prostate RCT [28] were eligible if patients were in one of three phases: newly diagnosed, biochemical recurrence, or advanced disease. Caregivers were spouses/partners who were ≥ 18 years old and lived with the patient. Patient-caregiver dyads in the Advanced Cancer RCT [29] were eligible if patients were diagnosed with stage III or IV lung, colorectal, breast or prostate cancer. Caregivers (e.g., spouses, siblings, adult children) were eligible if they were ≥ 18 years old and were identified by the patient as their primary caregiver. In both RCTs, family caregiver was defined as the primary person who provided emotional and/or physical care to the person with cancer as indicated by the cancer patient.

### Procedures

In both RCTs, clinic staff approached eligible participants at participating cancer centers. Dyads willing to participate were contacted by research staff and scheduled to complete baseline questionnaires at home. All participants provided written informed consent prior to data collection in their homes. Patients and caregivers completed questionnaires independently (often in separate rooms in their homes) in the presence of a data collector. Both RCTs were approved by the Institutional Review Boards at the University of Michigan and multiple cancer centers serving as recruitment sites. Study procedures were published previously [28, 29].

### Instruments

#### Caregiver QOL

The caregiver QOL measure was developed from the patient FACT-G (version 4), a multi-dimensional, cancer-specific scale developed in 1993 to measure patient QOL [17]. Items for the original patient FACT-G were initially developed using semi-structured feedback from cancer patients and oncology specialists [17]. The patient FACT-G consists of 27 Likert items measuring overall QOL and four subscales—physical, social, emotional, and functional well-being. Items are summed within each domain to obtain a subscale score and summed overall to provide a total score, with a range of 0–108 points. Higher scores indicate better QOL. Evidence of the reliability and validity of the patient version of the FACT-G is well-established [17] and the scale has been used widely.

As mentioned previously, to measure caregivers’ physical, social, emotional, and functional well-being and overall QOL in the context of cancer, three content experts made minor wording modifications to the original Patient FACT-G, enabling caregivers to report how the cancer was affecting their overall QOL and well-being on the four subscales. The Caregiver FACT-G consisted of 27 items and had the same four domains as the Patient FACT-G. The content validity of FACT-G was evaluated through focus groups (*N* = 6) conducted with 22 men with prostate cancer and 20 spouse caregivers [25, 26]. The questionnaires (including FACT-G) were evaluated for their difficulty, participants’ understanding of items, and time for completion (unpublished work). Our results from these preliminary studies revealed that the majority of items on the Patient FACT-G were also appropriate for the caregivers. The Caregiver FACT-G (Table 1) was scored in the same manner as the patient version, with responses ranging from not at all (0) to very much (4) and total scores ranging from 0 to 108. Table 1 also indicated the items that need to be reverse scored. During administration of the instrument, caregivers were instructed to take the patients’ illness into account when answering the questions.

**Table 1:** Caregiver FACT-G

		Not at all	A little bit	Somewhat	Quite a bit	Very much
Physical well-being						
PW1F1^#^	I have a lack of energy	0	1	2	3	4
PW1F2^#^	I have nausea	0	1	2	3	4
PW1F3^#^	Because of my physical condition, I have trouble meeting the needs of my family	0	1	2	3	4
PW1F4^#^	I have pain	0	1	2	3	4
PW1F5^#^	Are you currently taking any medication or receiving other medical treatment? No Yes, if yes, I am bothered by the side effects of my treatment	0	1	2	3	4
PW1F6^#^	I feel ill	0	1	2	3	4
PW1F7^#^	I am forced to spend time in bed	0	1	2	3	4
Social/family well-being						
SW1F1	I feel close to my friends	0	1	2	3	4
SW1F2	I get emotional support from my family	0	1	2	3	4
SW1F3	I get support from my friends	0	1	2	3	4
SW1F4	My family has accepted the illness	0	1	2	3	4
SW1F5	I am satisfied with family communication about the illness	0	1	2	3	4
SW1F6	I feel close to my partner (or the person who is my main support)	0	1	2	3	4
Regardless of your current level of sexual activity, please answer the following question. If you prefer not to answer it, check this box and go to the next section						
SW1F7	I am satisfied with my sex life	0	1	2	3	4
Emotional well-being						
EW1F1^#^	I feel sad	0	1	2	3	4
EW1F2	I am satisfied with how I’m coping with my family member’s illness	0	1	2	3	4
EW1F3^#^	I am losing hope in the fight against my family member’s illness	0	1	2	3	4
EW1F4^#^	I feel nervous	0	1	2	3	4
EW1F5^#^	I worry about my family member dying	0	1	2	3	4
EW1F6^#^	I worry that my family member’s condition will get worse	0	1	2	3	4
Functional well-being						
FW1F1	I am able to work (include work at home)	0	1	2	3	4
FW1F2	My work (include work at home) is fulfilling	0	1	2	3	4
FW1F3	I am able to enjoy life	0	1	2	3	4
FW1F4	I have accepted my family member’s illness	0	1	2	3	4
FW1F5	I am sleeping well	0	1	2	3	4
FW1F6	I am enjoying the things I usually do for fun	0	1	2	3	4
FW1F7	I am content with the quality of my life right now	0	1	2	3	4

Below is a list of statements that other people facing an illness have said are important. Please circle or mark one number per line to indicate your response as it applies to the *past 7 days*

Items with # need to be reverse scored

#### Validity testing

We used the same measures in both RCTs to assess the concurrent construct validity of the Caregiver FACT-G, with each scale having prior evidence of reliability and validity. The Transactional Theory of Stress [19, 30] guided our selection of factors used to examine the scale’s construct validity (convergent and discriminant) and predictive validity. We used hypothesis testing to examine the validity of Caregiver FACT-G, as suggested by Henrica de Vet et al. [32].

To examine convergent validity, we hypothesized that self-efficacy, active coping, dyadic communication, and social support would be positively associated with Caregiver FACT-G scores. Self-efficacy was measured with the 17-item Lewis Cancer Self-efficacy Scale [33]. Active coping was derived from items on the Brief Cope Scale [34]. Dyadic communication was measured with the 23-item Lewis Mutuality and Interpersonal Sensitivity Scale [33]. Social support was measured with the 7-item family support subscale of the Social Support Questionnaire [35].

To test discriminant validity, we hypothesized that appraisal of caregiving, uncertainty, hopelessness and avoidant coping would be negatively associated with Caregiver FACT-G scores. Appraisal of Caregiving was measured with the 32-item scale [36, 37]. Uncertainty was measured with the 28-item Mishel Uncertainty in Illness Scale [38] in the Prostate RCT and a brief nine-item version of the scale [38] in the Advanced Cancer RCT. Hopelessness was measured with the 20-item Beck Hopelessness Scale [39]. Avoidant coping was measured using items from the Brief Cope Scale [34].

To assess predictive validity, we hypothesized caregivers’ physical and mental well-being would be positively associated with their FACT-G scores, whereas depression would be negatively associated with their FACT-G scores. The 12-item Medical Outcomes Study Short Form Health Survey (SF-12, version 2) [40] has two dimensions: physical and mental well-being. We collected data on the SF-12 from the Prostrate RCT at 4 month follow-up. The Center for Epidemiological Studies-Depression Scale (CESD) [41] was used in the Advanced Cancer RCT at 3 months follow-up.

### Data analysis

We used SAS software (SAS 9.4, Cary, NC) to conduct the data analysis on each of the RCTs separately. We used the Consensus-based Standards for the Selection of Health Measurement Instruments checklist [42] to guide the evaluation of the Caregiver FACT-G.

To evaluate acceptability and precision of the Caregiver FACT-G, we calculated the percentage of missing data, floor and ceiling effects (participants’ lowest and highest responses, respectively) at the item level, and item-total correlations. Using confirmatory factor analysis (CFA), we examined the four-factor structure of the FACT-G as proposed by Cella and colleagues for the patient version [17]. We examined global model fit [43] using these statistics: *χ*^2^, comparative fit index (CFI), Tucker Lewis index (TLI), the root mean square error of approximation (RMSEA), and weighted root mean square residual [44]. For internal consistency testing, we examined Cronbach’s alpha coefficients and correlations between the four subscales and the total scale. Based on Cella et al.’s findings of high inter-correlations among FACT-G subscales [17], the FACT-G factors were assumed to be related and allowed to correlate. Finally, we conducted Pearson r correlation analyses to examine construct and predictive validity of the Caregiver FACT-G with the instruments listed earlier.

## Results

### Participant characteristics

The mean age of caregivers in the Prostate RCT was 59 years (SD = 9.1; range = 34–84). The mean education was 15 years (SD = 2.68; range = 8–22) and the median family income was $50,000-$75,000. Caregivers were predominantly White (83%) and African-American (13%), or another race/ethnicity (4%). Approximately 25% of the caregivers had health problems (e.g., arthritis, back problems). Patients had newly diagnosed prostate cancer (65%) who underwent prostectomy (60%) or received external beam radiation (40%); biochemical recurrent cancer (14%) under observation (50%) or treatment (primarily hormones) (50%); and advanced cancer (21%) receiving hormones (36%) and hormone-refractory treatments (e.g., chemotherapy) (64%).

The mean age of caregivers in the Advanced Cancer RCT was 56.7 years (SD = 12.6; range = 18–88), and mean education was 14.8 years (SD = 2.7; range = 18–22). Most were White (82.5%) and African-American (13.5%). Most caregivers were female (55.8%) and spouses (74%); 66% had health comorbid conditions (e.g., hypertension, heart problems). Patients had advanced lung (32.4%), colorectal (25.4%), breast (29.1%), or prostate cancer (13.0%) who were on an average 47 months since original diagnosis. Patients were currently receiving chemotherapy (66%), hormones (23%), radiation (8%), surgery (4%), or watchful waiting (6%) (multiple responses were possible).

### Acceptability and precision

#### Prostate RCT

The mean total Caregiver FACT-G score was 87.95 (SD = 12.9) (Table 2). The mean subscale scores were: Physical well-being, 25.1 (SD = 3.4); Social well-being, 22.4 (SD = 4.5); Emotional well-being, 18.4 (SD = 4.6); and Functional well-being, 22.0 (SD = 4.7). Rates of missing data ranged from 0% to 24.71%, with highest rates of missing data on items pertaining to caregivers’ physical well-being, i.e., side effects (24.7%) and satisfaction with sex life (22.4%). Floor effects varied from 0 to 21.57% with the highest percentage occurring among items in the social well-being subscale. Ceiling effect varied from 14.71 to 89.69% with the highest percentage occurring among items in the physical well-being subscale. Item-total correlations—relationships between individual items and the total FACT-G score—ranged from 0.29 to 0.70. A few physical well-being items (e.g., feeling ill or remaining in bed) had weak item-total correlations ranging 0.25–0.30.

**Table 2:** Caregiver FACT-G acceptability, precision and item-total correlation: prostate cancer and advanced cancer studies

Subscales and items	Prostate cancer study							Advanced cancer study						
	*N*	Mean	SD	% missing	% at floor	% at ceiling	Item-total	*N*	Mean	SD	% missing	% at floor	% at ceiling	Item-total
Physical well-being subscale	263	25.05	3.41	0.00	NA	NA	NA	484	24.02	4.15	0.00	NA	NA	NA
PW1F1	262	2.93	1.05	0.38	1.91	37.79	0.62	484	2.49	1.15	0.00	6.20	21.49	0.61
PW1F2	263	3.86	0.47	0.00	0.38	89.35	0.35	484	3.78	0.65	0.00	0.83	86.16	0.35
PW1F3	263	3.65	0.77	0.00	0.76	78.33	0.48	482	3.58	0.85	0.41	1.24	75.93	0.46
PW1F4	263	3.27	0.99	0.00	1.52	55.13	0.44	477	3.25	1.07	1.45	3.14	56.39	0.45
PW1F5	198	3.74	0.64	24.71	0.00	82.32	0.36	330	3.34	1.10	31.82	3.64	67.27	0.25
PW1F6	262	3.76	0.57	0.38	0.00	82.06	0.29	483	3.66	0.73	0.21	0.62	78.67	0.52
PW1F7	262	3.86	0.46	0.38	0.38	89.69	0.30	482	3.84	0.55	0.41	0.62	90.46	0.27
Social well-being subscale	263	22.44	4.49	0.00	NA	NA	NA	484	19.91	5.72	0.00	NA	NA	NA
SW1F1	263	3.16	0.97	0.00	1.52	47.53	0.52	484	2.75	1.11	0.00	4.13	29.34	0.56
SW1F2	263	3.37	0.89	0.00	1.52	57.41	0.52	483	2.91	1.13	0.21	2.90	40.58	0.52
SW1F3	263	3.22	0.90	0.00	0.76	47.91	0.44	484	2.88	1.12	0.00	3.31	37.19	0.51
SW1F4	261	3.51	0.74	0.76	0.38	63.22	0.48	483	3.04	0.97	0.21	1.24	38.92	0.49
SW1F5	262	3.31	0.89	0.38	0.76	53.44	0.56	484	2.83	1.19	0.00	5.99	37.40	0.48
SW1F6	263	3.62	0.77	0.00	1.14	74.90	0.42	477	3.39	0.96	1.45	2.52	62.89	0.36
SW1F7	204	1.92	1.34	22.43	21.57	14.71	0.38	365	1.82	1.45	24.59	28.49	16.71	0.33
Emotional well-being subscale	263	18.44	4.62	0.00	NA	NA	NA	484	13.99	5.17	0.00	NA	NA	NA
EW1F1	263	3.10	0.97	0.00	1.52	41.44	0.63	484	2.57	1.14	0.00	6.40	22.93	0.62
EW1F2	263	3.07	1.00	0.00	3.80	39.54	0.44	484	2.51	1.02	0.00	4.96	16.12	0.56
EW1F3	262	3.74	0.78	0.38	2.67	86.64	0.40	484	3.29	1.09	0.00	3.51	61.36	0.54
EW1F4	263	3.27	0.98	0.00	0.76	55.13	0.59	483	2.76	1.21	0.21	6.83	32.92	0.51
EW1F5	262	2.74	1.23	0.38	7.63	33.21	0.50	484	1.46	1.35	0.00	35.12	9.09	0.38
EW1F6	263	2.54	1.23	0.00	9.51	24.71	0.50	484	1.40	1.36	0.00	35.54	9.71	0.39
Functional well-being subscale	263	22.02	4.69	0.00	NA	NA	NA	484	18.56	5.71	0.00	NA	NA	NA
FW1F1	263	3.47	0.89	0.00	2.66	64.64	0.59	482	3.15	1.10	0.41	4.56	50.00	0.42
FW1F2	263	3.14	0.94	0.00	1.90	42.97	0.45	479	2.73	1.15	1.03	5.85	30.69	0.56
FW1F3	263	3.33	0.82	0.00	0.76	50.57	0.69	484	2.77	1.04	0.00	2.27	29.75	0.70
FW1F4	263	3.41	0.75	0.00	0.00	54.75	0.51	482	2.98	0.97	0.41	1.87	36.51	0.42
FW1F5	263	2.68	1.10	0.00	3.04	28.52	0.55	484	2.24	1.22	0.00	9.92	18.39	0.50
FW1F6	262	3.02	0.99	0.38	2.29	37.02	0.68	484	2.37	1.22	0.00	8.26	21.49	0.75
FW1F7	263	2.97	0.95	0.00	1.90	33.84	0.70	484	2.32	1.22	0.00	9.09	20.45	0.74
Caregiver FACT-G total	263	87.95	12.86	0.00	NA	NA	NA	484	76.49	15.81	0.00	NA	NA	NA

*PW* physical well-being, *FW* social well-being, *EW* emotional well-being, *FW* functional well-being, *Item-total* correlation with total (standardized)

#### Advanced cancer RCT

The mean total Caregiver FACT-G score was 76.49 (SD = 15.8) (Table 2). Mean subscale scores were: Physical well-being, 24.0 (SD = 4.2); Social well-being, 19.9 (SD = 5.7); Emotional well-being, 14.0 (SD = 5.2), and Functional well-being, 18.6 (SD = 5.7). Rates of missing items ranged from 0 to 31.82% with the highest rates occurring in questions related to caregivers’ concerns about physical well-being side effects (31.8%) and satisfaction with sex life (24.6%). Floor effects varied from 0.62 to 35.54% with the highest percentage occurring among items in the emotional well-being subscale, and ceiling effects varied from 9.09 to 90.46% with the highest percentage occurring among items in the physical well-being subscale. Item-total correlations ranged from 0.25 to 0.75.

### Factor structure: confirmatory factor analysis (CFA)

#### Prostate RCT

The CFA revealed moderate to high (standardized) factor loadings for physical (0.51–0.72), social (0.38–0.85), emotional (0.49–0.79) and functional well-being (0.54–0.86).

#### Advanced cancer RCT

The CFA revealed moderate to high (standardized) factor loadings for physical well-being (0.49–0.83), social well-being (0.31–0.84), emotional well-being (0.54–0.82) and functional well-being (0.43–0.87).

The global model fit results of both RCTs are in Table 3. The 4-factor solution was used for all subsequent analyses.

**Table 3:** Confirmatory factor analysis: prostate cancer and advanced cancer studies (Spearman correlation)

Global model fit	Prostate cancer study	Advanced cancer study
*χ*^2^-test for discrepancy between sample and fitted covariance matrices	*χ*^2^ = 699, df = 318, *p* < 0.001	*χ*^2^ = 999, df = 318, *p* < 0.001
Comparative fit index (CFI)	0.83	0.82
Tucker Lewis index (TLI)	0.81	0.80
Root mean square error of approximation (RMSEA)	0.078 (90% CI 0.07–0.09)	0.08 (90% CI 0.07–0.09)
Weighted standardized root mean square residual index	0.089	0.086

### Internal consistency testing

#### Prostate RCT

Cronbach’s alphas ranged from 0.81 to 0.91 for subscale and total reliability scores of the Caregiver FACT-G, indicating satisfactory (> 0.80) internal consistency (Table 4). The correlation coefficients among the physical, social, emotional, and functional subscales ranged from 0.24 to 0.51 (*p* < 0.001), indicating small to moderate inter-factor correlations using Cohen’s reference values [45]. The correlations between the total FACT-G and the subscales were moderate to large, ranging from 0.62 to 0.83 (*p* < 0.001).

**Table 4:** Internal consistency reliability and inter-factor correlation

Caregiver FACT-G subdomain	Prostate cancer study (*N* = 263)					Advanced cancer study (*N* = 484)				
	Cronbach's alpha	Inter-factor correlation				Cronbach's alpha	Inter-factor correlation			
		PW	SW	EW	FW		PW	SW	EW	FW
PW	0.81	1.00	0.24***	0.33***	0.40***	0.83	1.00	0.26***	0.31***	0.48***
SW	0.82	0.24***	1.00	0.40***	0.51***	0.84	0.26***	1.00	0.38***	0.56***
EW	0.83	0.33***	0.40***	1.00	0.49**	0.81	0.31***	0.38***	1.00	0.57***
FW	0.86	0.40***	0.51***	0.49***	1.00	0.84	0.48***	0.55***	0.57***	1.00
Total score	0.91	0.62***	0.74***	0.76***	0.83***	0.91	0.63***	0.76***	0.75***	0.87***

*FACT-G* Function Assessment of Cancer Therapy-General, *PW* physical well-being, *SW* social well-being, *EW* emotional well-being, *FW* functional well-being

**p < .001; ***p < .0001

#### Advanced cancer RCT

Cronbach’s alphas ranged from 0.81 to 0.91 for total and subscales reliabilities of the Caregiver FACT-G, indicating satisfactory (> 0.80) internal consistency. The correlations among the physical, social, emotional, and functional subscales ranged from 0.26 to 0.57 (*p* < 0.001), indicating small to moderate relationships. The correlations between the total FACT-G scale and the subscales were moderate to large, ranging from 0.63 to 0.87 (*p* < 0.001) (Table 4).

### Validity testing

#### Prostate RCT

Mean Caregiver FACT-G subscale and total scores were positively correlated with self-efficacy, dyadic communication, and social support, indicating satisfactory convergent validity (Table 5). There were mixed findings, however, with active coping variable: more active coping was related with higher social well-being but with lower physical and emotional well-being. The mean FACT-G subscale and total scores were negatively correlated with appraisal of caregiving, uncertainty, hopelessness, and avoidant coping, indicating satisfactory discriminant validity.

**Table 5:** Validity testing: prostate cancer and advanced cancer study (spearman correlation with 95% CI)

Caregiver FACT-G validity	Prostate cancer study (N = 263)					Advanced cancer study (N = 484)								
	PW	SW	EW	FW	Total	PW		SW		EW		FW		Total
Convergent														
Self-efficacy scale	.32*** (.21,.43)	.51*** (.41,.59)	.54*** (.44,.62)	.51*** (.42,.60)	.63*** (.55,.69)	.25*** (.16,.33)		.46*** (.38,.53)		.48*** (.41,.55)		.49*** (.41,.55)		.55*** (.49,.61)
Active coping	− .22*** (− .33, − .10)	.15* (.03,.27)	− .23*** (− .34, − .11)	− .03 (− .15,.09)	− .10 (− .22,.02)	− .05 (− .14, .04)		.31*** (.23,.39)		− .05 (− .14, .04)		.08 (− .01, .16)		.10* (.01, .20)
Dyadic communication	.29*** (.17,.40)	.51*** (.41,.59)	.33*** (.22,.43)	.36*** (.25,.46)	.49*** (.39,.58)	0.18*** (.09, .26)		0.43*** (.35, .50)		0.32*** (.24, .40)		0.35*** (.27, .42)		0.42*** (.34, .49)
Social support	0.18* (.06, .30)	0.51*** (.42, .60)	0.22** (.10, .33	0.41*** (.31, .51)	0.44*** (.33, .53)	0.27*** (.19, .35)		0.53*** (.47, .60)		0.39*** (.31, .47)		0.46*** (.38, .52)		0.54*** (.47, .60)
Discriminant														
Appraisal of caregiving	− 0.37*** (− .47, − .26)	− 0.41*** (− .51, − .31)	− 0.60*** (− .67, − .52)	− 0.50*** (− .59, − .40)	− 0.63*** (− .69, − .55)	− .44*** (− .51, − .37)		− .49*** (− .56, − .42)		− .59*** (− .64, − .53)		− .60*** (− .65, − .54)		− .69*** (− .73, − .64)
Uncertainty scale	− .28*** (− .38, − .16)	− .38*** (− .47, − .27)	− .50*** (− .59, − .41)	− .47*** (− .56, − .39)	− .53*** (− .62, − .44)	− .37*** (− .44, − .29)		− .33*** (− .41, − .25)		− .51*** (− .57, − .44)		− .47*** (− .53, − .39)		− .53*** (− .60, − .47)
Hopelessness scale	− .26*** (− .37, − .15)	− .25*** (− .36, − .13)	− .42*** (− .52, − .32)	− .41*** (− .51, − .31)	− .45*** (− .54, − .34)	− .37*** (− .45, − .29)		− .42*** (− .49, − .35)		− .59*** (− .65, -.53)		− .49*** (− .56, − .42)		− .60*** (− .65, − .54)
Avoidant coping	− .41*** (− .50, − .30)	− .32*** (− .42, − .21)	− .58*** (− .65, − .49)	− .44*** (− .53, − .33)	− .58*** (− .65, − .49)	− .38*** (− .45, − .30)		− .26*** (− .34, − .18)		− .55*** (− .61, − .49)		− .47*** (− .54, − .40)		− .54*** (− .60, − .47)
Predictive														
SF-12 mental summary score	.40*** (.29, .50)	.34*** (.23, .45)	.40*** (.29, .50)	.48*** (.38, .57)	.54*** (.44, .62)	NA								
SF-12 physical summary score	.37*** (.25, .47)	− .05 (− .18, .08)	.15* (.02, .27)	.23** (.11, .35)	.23** (.10, .34)									
Depression CESD	NA					− .49*** (− .56, − .40)	− .46*** (− .53, − .37)		− .60*** (− .66, − .53)		− .61*** (− .67, − .54)		− .69*** (− .74, − .63)	

Spearman Correlation Coefficients, Prob >|*r*| under H0: ρ = 0

*FACT-G* function assessment of cancer therapy-general, *PW* physical well-being, *SW* social well-being, *EW* emotional well-being, *FW* functional well-being

*p < .05; **p < .001; ***p < .0001

The baseline Caregiver FACT-G total and four subscale scores were positively correlated with their SF-12 *mental* component scores at 4 months follow-up. Similarly, caregivers’ baseline FACT-G total and three subscale scores (physical, emotional, and functional) were positively correlated with higher SF-12 *physical* component scores at follow-up, indicating satisfactory predictive validity.

#### Advanced cancer RCT

The mean Caregiver FACT-G subscale and total scores were positively correlated with self-efficacy, dyadic communication, and social support, indicating satisfactory convergent validity (Table 5). Active coping was positively correlated with social support and the total FACT-G score. In addition, the mean FACT-G subscales and total scores were negatively correlated with appraisal of caregiving, uncertainty, hopelessness, and avoidant coping, indicating discriminant validity.

The baseline Caregiver FACT-G total and four subscale scores were negatively correlated with greater CESD scores at 3-month follow-up. The results indicated baseline FACT-G subscale and total scores predicted worse depression among caregivers of advanced cancer patients at T2 follow-up.

## Discussion

Demand is growing for caregivers to deliver high quality care to cancer patients at home. Thus, there is a critical and immediate need to use established instruments to assess the effect of these demands on caregivers’ QOL. To address this need, we conducted a comprehensive assessment of the Caregiver FACT-G using the data obtained from two large RCTs with cancer patients and their caregivers.

### Psychometric properties of the caregiver FACT-G

Overall the Caregiver FACT-G scale was acceptable and precise in measuring caregiver QOL. However, some of the items (e.g., treatment side effects, feeling ill, remaining in bed in the physical well-being subscale and sex life in the social well-being subscale) had weak item-total correlations and/or high ceiling effects, and thus, may need refinement. CFA results indicated high to moderate factor loadings for physical, social, emotional, and functional well-being on the total score, consistent with the factor structure reported for the widely-used patient FACT-G [17]. The high inter-factor correlations, with the Cronbach alphas ranging from 0.81–0.91, indicated the Caregiver FACT-G has high internal consistency.

Our findings also indicated that the Caregiver FACT-G has strong convergent and discriminant validity, consistent with expected relationships within the Transactional Theory of Stress [19]. Regarding convergent validity, our results indicated that higher caregiver self-efficacy, social support, and dyadic communication were significantly associated with higher Caregiver FACT-G total and subscale scores; however, findings were mixed with the variable active coping. Specifically, while active coping was positively correlated with caregivers’ FACT-G social well-being in both RCTs and with the FACT-G total score in the Advanced Cancer RCT, active coping was not consistently related to other FACT-G subscales. Other studies have also reported mixed findings between QOL and active coping [46–48], suggesting that this variable warrants further exploration. We also found strong discriminant validity of the Caregiver FACT-G. The total and subscale scores of the Caregiver FACT-G were consistently related to negative caregiving appraisal, uncertainty, hopelessness and avoidance coping. These findings are consistent with those from previous research [7, 30].

One of the most important findings of the study was the strong evidence of the predictive validity of the Caregiver FACT-G. In the Prostate RCT, the Caregiver FACT-G measured at baseline predicted which caregivers had lower physical and mental well-being 4-months later. Similarly, the Caregiver FACT-G predicted which caregivers in the Advanced Cancer RCT study were likely to have higher depression levels 3 months later. These results, confirming our hypotheses, indicated that the Caregiver FACT-G has strong predictive validity among male and female caregivers with varying demographic characteristics, and among caregivers of patients who varied in the stage and type of cancer and health history. Given its predictive validity, the Caregiver FACT-G could be a useful instrument for identifying caregivers at risk of poorer outcomes in future studies and facilitating early intervention for those caregivers at high risk.

Caregivers in the Prostate RCT reported higher QOL than caregivers in the Advanced Cancer RCT. This was not surprising because most of the caregivers in the Prostate RCT was caring for men with localized cancer, while all caregivers in the Advanced RCT were caring for patients with metastatic disease. These findings demonstrated the ability of the Caregiver FACT-G to detect the differences in QOL among caregivers of patients with different stages of cancer.

### Limitations and future direction

Although we found strong evidence for the reliability and validity of the Caregiver FACT-G, a few limitations need to be addressed in future research. First, we modified the Patient FACT-G to obtain the Caregiver FACT-G based on our preliminary studies, focus groups of cancer patients and caregivers, and expert feedback. Future assessment of the Caregiver FACT-G would benefit from studies using cognitive interviewing techniques, such as think-aloud, verbal probing, paraphrasing and response latencies [49] to refine the instrument. Second, some of the items had more missing data than others, such as the item about caregivers’ satisfaction with their sex life. Data on this item were also missing on the Patient FACT-G when administered previously [17], suggesting patients’ and caregivers’ preference for privacy on this topic. Future research can explore reasons for the missing data and consider alternative ways to assess their sexual satisfaction. Third, we found a lower item-total correlation and more ceiling effects with three items on caregivers’ physical well-being subscale (i.e., side effects, feel ill, spend time in bed), even though 25% and 66% of the caregivers in the Prostate and Advanced Cancer RCTs, respectively, reported health problems of their own. Caregivers often ignore their own physical health needs and focus solely on the needs of the patient. While some caregivers reported they lacked energy (PW1F1), almost none reported spending time in bed (PW1F7), possibly because caregivers were not ill or they had caregiving responsibilities. Qualitative research with caregivers can help to improve the wording or relevance of the items on the physical well-being subscale. Another limitation was related to the sub-optimal CFA model fit according to the recommended threshold [43, 44]. Although it is not uncommon to see sub-optimal model fit [50], especially for some indices, results suggest that some items of the caregiver FACT-G (e.g., the physical well-being items) may need further evaluation. On the other hand, the strong evidence of the validity of caregiver FACT-G indicated that it holds promise as a measure of caregiver QOL. Finally, the majority of the caregivers in our RCTs were spouses. Future research is needed to examine whether caregivers who do not share a home with the patient are affected differently by the patients' disease.

### Implications of findings

The Caregiver FACT-G demonstrated promising psychometric properties in measuring caregiver QOL among two large samples of caregivers of patients with different types and stages of cancer. With parallel versions of the FACT-G for both patients and caregivers, researchers can compare patient and caregiver QOL as well as determine the extent to which caregiver and patient QOL are interrelated and influence one another. In view of the limited tools to assess caregiver QOL in family and cancer research, this research supports the use of the Caregiver FACT-G to assess caregivers’ QOL. The measure can determine which caregivers are at higher risk of poorer QOL over time, as well as to test the effect of dyadic interventions on caregiver and patient outcomes.
